# Enhancing the thermostability of α-L-rhamnosidase from *Aspergillus terreus* and the enzymatic conversion of rutin to isoquercitrin by adding sorbitol

**DOI:** 10.1186/s12896-017-0342-9

**Published:** 2017-02-27

**Authors:** Lin Ge, Anna Chen, Jianjun Pei, Linguo Zhao, Xianying Fang, Gang Ding, Zhenzhong Wang, Wei Xiao, Feng Tang

**Affiliations:** 1 Co-Innovation Center for Sustainable Forestry in Southern China, Nanjing Forestry University, 159 Long Pan Road, Nanjing, 210037 China; 2 College of Chemical Engineering, Nanjing Forestry University, 159 Long Pan Road, Nanjing, 210037 China; 3 Jiangsu Kanion Pharmaceutical Co., Ltd, 58 Haichang South Road, Lianyungang, 222001 Jiangsu Province China; 4 International centre for bamboo and rattan, 8 FuTong East Street, Beijing, 100714 China

**Keywords:** Thermostability, α-L-Rhamnosidase, Sorbitol, Enzymatic conversion

## Abstract

**Background:**

Thermally stable α-L-rhamnosidase with cleaving terminal α-L-rhamnose activity has great potential in industrial application. Therefore, it is necessary to find a proper method to improve the thermal stability of α-L-rhamnosidase.

**Results:**

In this study, addition of sorbitol has been found to increase the thermostability of α-L-rhamnosidase from *Aspergillus terreus* at temperatures ranging from 65 °C to 80 °C. Half-life and activation free energy with addition of 2.0 M sorbitol at 70 °C were increased by 17.2-fold, 8.2 kJ/mol, respectively. The analyses of the results of fluorescence spectroscopy and CD have indicated that sorbitol helped to protect the tertiary and secondary structure of α-L-rhamnosidase. Moreover, the isoquercitrin yield increased from 60.01 to 96.43% with the addition of 1.5 M of sorbitol at 70 °C.

**Conclusion:**

Our findings provide an effective approach for enhancing the thermostability of α-L-rhamnosidase from *Aspergillus terreus*, which makes it a good candidate for industrial processes of isoquercitrin preparation.

**Electronic supplementary material:**

The online version of this article (doi:10.1186/s12896-017-0342-9) contains supplementary material, which is available to authorized users.

## Background

Isoquercitrin, a derhamnosylation product of rutin, has been widely acknowledged with several biological activities, including anti-mutagenesis, anti-virus, anti-hypertensive, anti-proliferative effects, lipid peroxidation, oxidative-stress protection as well as other pharmacological effects [1–4]. Isoquercitrin recently has been proved to exhibit better bioactivity than rutin [5]. However, isoquercitrin is rarely found in nature, and therefore it has hardly been isolated. Isoquercitrin is structurally similar to rutin that is abundantly present in plant [6]. Therefore, rutin can be an ideal precursor for preparing isoquercitrin. So far, several methods for the transformation of rutin to isoquercitrin have been investigated, including acid hydrolysis, heating, microbial transformation, and enzymatic transformation techniques [7–9] the last of which has become the favored method due to its economic merits, eco-friendly status and applicability to the food industry.

The enzyme α-L-rhamnosidase (RASE, E.C. 3.2.1.40), which is able to release terminal α-L-rhamnose exclusively from glycosides, glycolipids, and other natural products [10–14], is widely distributed in nature and has been purified from animal tissues, plants, yeasts, fungi and bacteria [15–22]. This enzyme as catalyst has been generally used in a large number of industrial applications. For instance, removing naringin that is the bitter component of grapefruit juices and other citrus juices [23], enhancing the aroma of wines and grape juice [11], studying of the structure of plants and bacterial polysaccharides [24], producing of rhamnose from various natural products [25]. Furthermore, α-L-rhamnosidase is used not only for the derhamnosylation of natural compounds but also for the reverse rhamnosylation of various small organic compounds [26].

However, the majority of the reported α-L-rhamnosidases are not thermally stable at higher temperatures [21, 27–29], which limits their industrial use. Therefore, it is necessary to find a proper method to improve the thermal stability of α-L-rhamnosidase with potential industrial roles. The thermostability of an enzyme hinges on multifarious causes, including amino acid composition, metal ions, pH as well as others [30–32]. Currently the thermostability of enzymes can be improved by chemical modification, immobilization, treatment with additives and protein engineering [33–36]. Among them, the effects of additives on the thermostability of the enzyme has received increasing attention from researchers because addition of additives to protein solution and changing its microenvironment provides a simple but practical means of increasing the stability of the enzyme [33]. Polyhydroxy compound sorbitol is widely used as the enzyme stabilizer [33, 37, 38]. Different mechanisms have been attributed to the stabilizing effect of sorbitol, which include preferential hydration, preferential exclusion from the denatured protein, and coating effect [39–41]. However, the effect of sorbitol at the molecular level is not known in detail. It is generally believed that the improvement of the thermostability of the enzyme by sorbitol is due to solvophobic interactions and hydrogen bonds [33].

Enhancement of thermal stability is beneficial for most of the biotechnological applications of proteins. However, the recombinant α-L-rhamnosidase of *Aspergillus terreus* has relatively low thermostability (half-life < 30 min at 70 °C) [42], which severely limits its industrial application. Naturally occurring osmolytes such as amino acids, polyols and salts are known to protect proteins against thermal inactivation by stabilizing the thermally unfolded proteins. In the present investigation, the effect of sorbitol on the thermostability of α-L-rhamnosidase was studied, and stability properties of the enzyme in the absence and the presence of sorbitol was determined, and the mechanisms responsible for sorbitol improving the thermostability of the enzyme were analyzed. Moreover, the effect of sorbitol on the enzymatic conversion of rutin to isoquercitrin was investigated. To the best of our knowledge, no report has been published concerning the application of polyhydroxy compound for improving the thermostability of the enzyme so as to enhance the enzymatic conversion of rutin to isoquercitrin.

## Methods

### Bacterial strains and plasmids

The gene encoding α-L-rhamnosidase (NCBI accession number JN899401.1) from *A. terreus* (Rha) was optimized based on the preferred codon usage of *P. pastoris* and synthesized by Shanghai Generay Biotech Co. Ltd. (Shanghai, China). The synthetic gene (MRha) was inserted into plasmid pPICZαA (Invitrogen, USA) to generate the expression vector pPICZαA/MRha, which were linearized with SacI and transformed into yeast strain *P. pastoris* KM71H (Invitrogen, USA) by electroporation. The sequence alignment of Rha and MRha was shown in Additional file 1: Figure S1.

### Production and purification of α-L-rhamnosidase

Yeast strain *P. pastoris* KM71H was cryopreserved at −80 °C in 15% (v/v) glycerol (OD_600 nm_ = 50–100), added into YPD (1% (w/v) yeast extract; 2% (w/v) peptone; 2% (w/v) glucose) medium, or kept on YPD plates (YPD medium with 2% (w/v) agar). For the *P. pastoris* cultivation the following media were used: YPD, BMGY (1% (w/v) yeast extract; 2% (w/v) peptone; 100 mM potassium phosphate, pH 6.0; 1.34% (w/v) YNB (Invitrogen, USA); 4 × 10-5% (w/v) biotin; 1% (v/v) glycerol). *P. pastoris* KM71H was cultivated on a rotary shaker at 28 °C and 180 rpm.

The mycelium was removed from the cultivation medium by filtration. The filtrate was precipitated with ammonium sulphate to 80% saturation. The enzyme precipitate was dissolved in 50 mM Tris-HCl buffer (pH 7.5) and the enzyme solution was dialyzed against 50 mM Tris-HCl buffer (pH 7.5) at 4 °C for 48 h. The dialysate was loaded onto a column of DEAE Sepharose Fast Flow (Amersham Bioscience, USA) equilibrated with 50 mM Tris-HCl buffer (pH 7.5). The enzyme was eluted from the column using linear gradient (20–300 mM NaCl). Fractions containing α-L-rhamnosidase were pooled and dialyzed against distilled water at 4 °C for 4 h and then against 10 mM phosphate buffer (pH 6.5) at 4 °C for 20 h. The purified enzyme was aliquotted and stored at −80 °C.

### Protein concentration determination

The protein concentration was quantified by the Bradford method [43], using the TaKaRa Bradford protein assay kit with bovine serum albumin (Dalian, TaKaRa Biotechnology, China) as the standard.

### Assay of α-L-rhamnosidase

α-L-Rhamnosidase activity was assayed using *p*NPR (Sigma Aldrich, USA) as substrate. One unit of enzymatic activity was defined as the amount of enzyme releasing 1 μmol of *p*-nitrophenol per minute in 100 mM citrate-phosphate buffer at pH 6.5 and 65 °C. After incubation of the reaction mixture at 65 °C for 10 min, the liberated *p*-nitrophenol was determined spectrophotometrically at 405 nm under alkaline conditions (100 μL of the reaction mixture was added to 300 μL of 1 M Na_2_CO_3_).

### Thermostability determination

The thermostability of α-L-rhamnosidase was determined by incubating the purified enzyme (1.13 mg/mL) in the presence and absence of sorbitol in 50 mM phosphate buffer (pH 6.5) at 70 °C for 1 h. And the samples were then centrifuged at 10000 rpm for 10 min. The residual α-L-rhamnosidase activity was determined as previously described. Measurements were performed in triplicate.

### Thermal stability profile

The purified enzyme (1.13 mg/mL) in the presence and absence of sorbitol was incubated at temperatures ranging from 60 to 85 °C in 50 mM phosphate buffer (pH 6.5). Aliquots were removed after 10 min of incubation, and the residual α-L-rhamnosidase activity was determined as previously described. The values of T_50_ for the presence and absence of sorbitol, defined as the temperature at which 50% of the initial activity was retained, were determined from the plots of residual activity (%) versus temperature. Measurements were performed in triplicate.

### Kinetics of thermal inactivation

The purified enzyme (1.13 mg/mL) in the presence and absence of sorbitol was incubated at temperatures ranging from 65 to 75 °C in 50 mM phosphate buffer (pH 6.5). Aliquots were removed at scheduled times, and the residual α-L-rhamnosidase activity was determined as previously described. Half-life was calculated from the first-order rate constants of inactivation, which were obtained from linear regression in logarithmic coordinates. The activation free energy (ΔG^≠^) was calculated as previously described [44]. Measurements were performed in triplicate.

### Kinetic parameters

The Michaelis-Menten parameters, *K*_*M*_ and *Vmax* were determined from Michaelis–Menten plots by measuring the initial reaction rates with different substrate concentrations at pH 6.5 and 65 °C. Measurements were performed in triplicate.

### Surface hydrophobicity analysis

The surface hydrophobicity (H_0_) of the purified enzyme was determined using a fluorescence probe called ANS (Sangon Biotech, Shanghai, China), as previously described [45]. According to the method previously described [46], protein concentrations were diluted (0.02, 0.04, 0.06, 0.08 and 0.1 mg/ml) in 10 mM phosphate buffer solution (pH 6.5). Then, aliquots (5 μL) of ANS (8.0 mM in the same buffer) were added to 1 ml of sample. The fluorescence intensity at 495 nm was measured using a Cell imaging multifunctional test system (BioTek, Cytation3, America) with excitation at 370 nm. H_0_ was calculated by linear regression analysis using the initial slope of the fluorescence intensity versus protein concentration plot. Measurements were performed in triplicate.

### Intrinsic fluorescence emission spectroscopy

Intrinsic fluorescence emission spectroscopy of α-L-rhamnosidase samples (0.1 mg/ml) in the presence and absence of sorbitol were measured in 10 mM phosphate buffer (pH 6.5) at room temperature using a fluorescence spectrophotometer (LS55, PE, America). To minimise the contribution of tyrosine residues to the emission spectra, the protein solutions were excited at 295 nm, and emission spectra were recorded from 320 to 400 nm at a scanning speed of 300 nm/min. The excitation and emission slit widths were 15 nm and 5 nm, respectively. All spectra were recorded in triplicate and corrected for the fluorescence of a protein-free sample.

### Analysis of CD spectrum

A MOS-450 CD spectrometer (Biologic, Claix, Charente, France) was used for CD analysis. The CD spectra with UV (190–240 nm) region were recorded with a 2 mm path-length cell at room temperature. The spectra were obtained as the average of four scans with a bandwidth of 0.1 nm, a step resolution of 1 nm and a scan rate of 1 nm/s. The CD spectra of α-L-rhamnosidase (0.1 mg/ml) in the presence and absence of sorbitol were recorded in 10 mM phosphate buffer (pH 6.5), and corrected by subtracting control spectra of protein-free buffer solutions. Analysis of the protein secondary structure was performed using Dichroweb [47]. Four secondary structures, α-helix, β-sheet, turn, and random coil, were calculated.

### Enzymatic hydrolysis of rutin and analysis of enzymatic hydrolysate

Rutin (Sangon Biotech, Shanghai, China) was treated with purified α-L-rhamnosidase, and the enzymatic hydrolysate was analyzed using an HPLC 1200 system (Agilent, USA) and a C18 column (4.6 × 250 mm; i.d. 5 μm; S.No. USAG008115, USA) with distilled water (A) and methanol (B) at A/B ratios 60:40 and run times of 30 min. The flow rate was 1.0 mL/min and the column was maintained at 30 °C, and detection was performed by monitoring the absorbance at 368 nm.

All enzymatic reactions were carried out in a temperature-controlled heating water bath. In this study, disodium hydrogen phosphate-citrate buffer (pH 4.5–6.5) were used. The typical reaction mixture (400 μL) contained disodium hydrogen phosphate-citrate buffer (pH 6.5), 8 mM of rutin and sorbitol. The reaction was started by adding the buffered solution of α-L-rhamnosidase from *Aspergillus terreus*, and the mixtures were incubated at 70 °C for different amounts of time at various sorbitol concentrations, pH values, substrate concentrations, enzyme concentrations while the other conditions were fixed in a temperature-controlled heating water bath. The reaction was stopped by adding 1 mL methanol. The crude hydrolysis products of rutin were then centrifuged at 10000 rpm for 10 min, and the supernatant solutions were filtered through a 0.45 μm filter before injection into the HPLC. Each value represents the mean of three independent measurements. The isoquercitrin yield and isoquercitrin concentration was calculated as follows.

$$ \mathrm{Isoquercitrin}\ \mathrm{yield}\kern0.75em \left(\%\right) = \frac{\mathrm{molar}\ \mathrm{amount}\ \mathrm{of}\ \mathrm{isoquercitrin}\ \left(\mathrm{mM}\right)}{\mathrm{initial}\ \mathrm{molar}\ \mathrm{amount}\ \mathrm{of}\ \mathrm{rutin}\ \left(\mathrm{mM}\right)} \times 100 $$

$$ \mathrm{Isoquercitrin}\ \mathrm{concentration}\ \left(\mathrm{mM}\right) = \mathrm{isoquercitrin}\ \mathrm{yield}\ \left(\%\right) \times \mathrm{initial}\ \mathrm{rutin}\ \mathrm{concentration}\kern0.5em \left(\mathrm{mM}\right) $$

## Results

### Sorbitol enhanced the thermostability of α-L-rhamnosidase

**Fig. 1:**
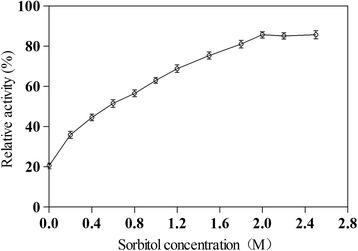
Effects of sorbitol, at different concentrations, on the thermostability of α-L-rhamnosidase at 70 °C. The level of thermostability before each incubation was defined as 100%. Each value represents the mean of three independent measurements

From the practical point of view, thermostability is one of the most important characteristics to be considered for applying enzymes in industrial processes [48]. Therefore, the effects of different concentrations of sorbitol on the thermostability of α-L-rhamnosidase were determined. As shown in Fig. 1, the thermostability of α-L-rhamnosidase was increased in the presence of sorbitol ranging in molar concentration from 0.2 M to 2.5 M after incubation at 70 °C for 1 h. When the sorbitol concentration was less than 2.0 M, the thermostability enhancement of α-L-rhamnosidase was significantly increased following the increase of the sorbitol concentration. However, when the sorbitol concentration was more than 2.0 M, the thermostability enhancement of α-L-rhamnosidase was almost constant, which increased 65.3% by comparison without sorbitol. It clearly illustrated that 2.0 M sorbitol concentration is a point of inflection in thermostability enhancement of α-L-rhamnosidase resulted from adding sorbitol, and also it is the maximum peak for the rate of rise based on the increase curve of the α-L-rhamnosidase thermostability, For this reason, our further studies were focused on the reaction system with adding 2.0 M sorbitol.

### Stability properties

**Fig. 2:**
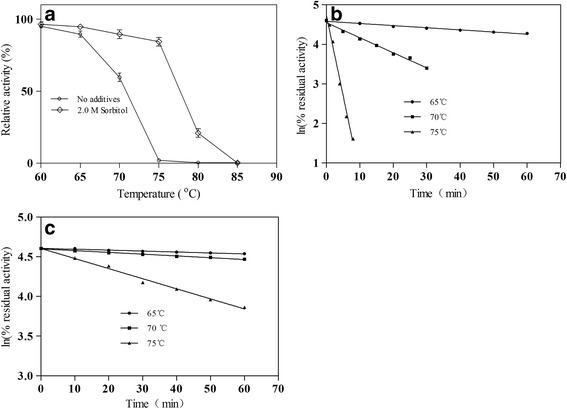
**a** Thermal stability profile of α-L-rhamnosidase with and without 2.0 M of sorbitol incubated at different temperatures ranging from 60 °C to 85 °C for 10 min. **b** The kinetics of thermal inactivation of α-L-rhamnosidase without sorbitol incubated at different temperatures ranging from 65 to 75 °C for several time intervals. **c** The kinetics of thermal inactivation of α-L-rhamnosidase with sorbitol incubated at different temperatures ranging from 65 to 75 °C for several time intervals. The level of thermostability before each incubation was defined as 100%. Each value represents the mean of three independent measurements

The thermal stability profile of the presence and absence of sorbitol was determined by incubation during 10 min at temperatures ranging from 60 to 85 °C. As illustrated in Fig. 2a, after addition of 2.0 M sorbitol, the thermostability of enzyme was increased at temperatures ranging from 60 to 85 °C. Consequently, the value of T_50_ was increased by about 7 °C for α-L-rhamnosidase after addition of 2.0 M sorbitol.

The kinetics of thermal inactivation of the absence of sorbitol was determined by incubating the enzyme at several temperatures ranging from 65 to 75 °C during 1 h (Fig. 2b). The kinetics of thermal inactivation of the presence of sorbitol was also determined by incubating the enzyme at several temperatures ranging from 65 to 75 °C during 1 h (Fig. 2c). The results showed that addition of 2.0 M sorbitol decreased thermal inactivation rates. The half-life of α-L-rhamnosidase was increased at each temperature after addition of 2.0 M sorbitol (Table 1), which prolonged the half-life at 65 °C, 70 °C and 75 °C by 4.5-fold, 17.2-fold and 30.3-fold, respectively. The activation free energy (ΔG^≠^) for thermal denaturation of α-L-rhamnosidase was increased at each temperature after addition of 2.0 M sorbitol (Table 1), which increased 4.2 kJ/mol, 8.2 kJ/mol and 10.0 kJ/mol of the activation free energy (ΔG^≠^) at 65 °C, 70 °C and 75 °C, respectively. Taking into account the enzyme in the practical application, 70 °C was chose in the following experiment.

**Table 1:** Half-life and activation free energy (ΔG^≠^) of α-L-rhamnosidase with and without 2.0 M of sorbitol

Temperature (°C)	Half-life (min)		ΔG^≠^ (kJ/mol)	
	Control^a, b^	2.0 M Sorbitol^a, b^	Control^a, b^	2.0 M Sorbitol^a, b^
65	127.9 ± 1	580.3 ± 4	86.3 ± 0.4	90.5 ± 0.6
70	18.2 ± 0.2	313.7 ± 2	82.0 ± 0.3	90.2 ± 0.8
75	1.8 ± 0.1	54.6 ± 0.4	76.5 ± 0.5	86.5 ± 0.6

^a^Control, α-L-rhamnosidase without 2.0 M sorbitol

^b^Values are the means ± SD (*n* = 3)

The kinetic parameters of the enzymes were analyzed using *p*NPR as a substrate at pH 6.5 and 65 °C. As shown Table 2, addition of sorbitol had a little effect on the values of *K*_*M*_ and *kcat/K*_*M*_.

**Table 2:** Kinetic parameters of α-L-rhamnosidase with and without sorbitol

Sample	Specific activity^A^ (U/mg)	*K*_*M*_^A^ (mM)	*k*_*cat*_^A^ (s^−1^)	*k*_*cat*_ */K*_*M*_ (s ^−1^M ^-1^)
No additives	451.5 ± 12.5	0.481 ± 0.01	407.6 ± 11.6	8.5 × 10^3^
0.6 M Sorbitol	414.5 ± 11.6	0.493 ± 0.02	379.2 ± 10.6	7.7 × 10^3^
1.0 M Sorbitol	354.4 ± 10.4	0.542 ± 0.02	362.5 ± 10.7	6.7 × 10^3^

^A^Values are the means ± SD (*n* = 3)

### Surface hydrophobicity analysis

The surface hydrophobicity of α-L-rhamnosidase was measured after incubation at 70 °C for 4 h. As shown Table 3, the addition of different concentrations of sorbitol noticeably affected the surface hydrophobicity of α-L-rhamnosidase. Compared to the absence of sorbitol, the addition of 0.6 M and 1.0 M of sorbitol increased the surface hydrophobicity of α-L-rhamnosidase by 23.5 and 27%, respectively.

**Table 3:** Surface hydrophobicity values of α-L-rhamnosidase with and without sorbitol before and after incubation

Samples	H_0_ values^c^
Control^a^	41483 ± 179.0
No additives^b^	289507 ± 112.1
0.6 M Sorbitol^b^	368005 ± 110.5
1.0 M Sorbitol^b^	357237 ± 238.1

^a^Control, α-L-Rhamnosidase before heating

^b^Samples after heating at 70 °C for 4 h

^c^Values are the means ± SD (*n* = 3)

### Emission fluorescence spectroscopic analysis

**Fig. 3:**
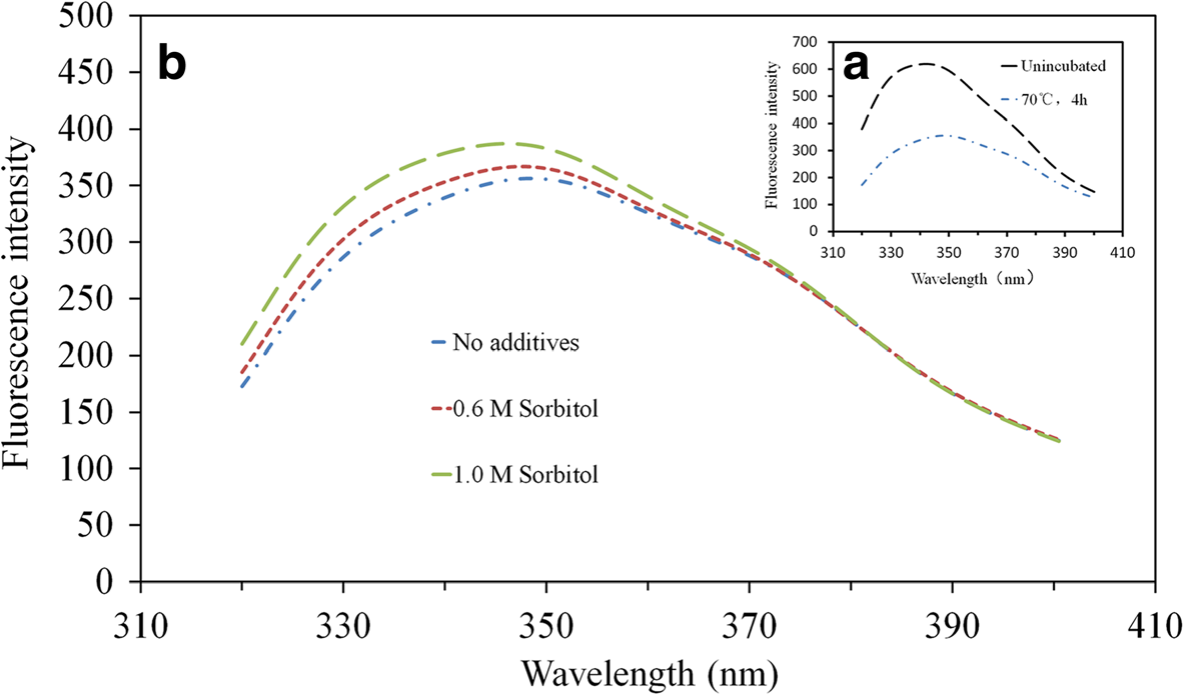
The intrinsic fluorescence spectra of α-L-rhamnosidase. **a** Without sorbitol before and after incubation at 70°C for 4 h.** b** Incubated at 70°C for 4 h with different concentrations of sorbitol in 10 mM phosphate buffer at pH 6.5. All spectra were corrected for the fluorescence of a protein-free sample. Each value represents the mean of three independent measurements

The fluorescence emission spectra of native α-L-rhamnosidase was examined. As shown in Fig. 3a, when excited at 295 nm, native α-L-rhamnosidase exhibited a maximum fluorescence emission at 342 nm. However, the maximum fluorescence intensity decreased rapidly, which was from 619.5 to 356.6 fluorescence intensity, and the fluorescence maximum exhibited a slight red shift, which was from 342 nm to 347 nm after incubation at 70 °C for 4 h. When sorbitol was added to the enzyme solution, the fluorescence intensity increased (Fig. 3b). Moreover, the fluorescence maximum showed a blue shift with the addition of sorbitol, which was from 348.5 nm to 346 nm.

### CD spectroscopy

**Fig. 4:**
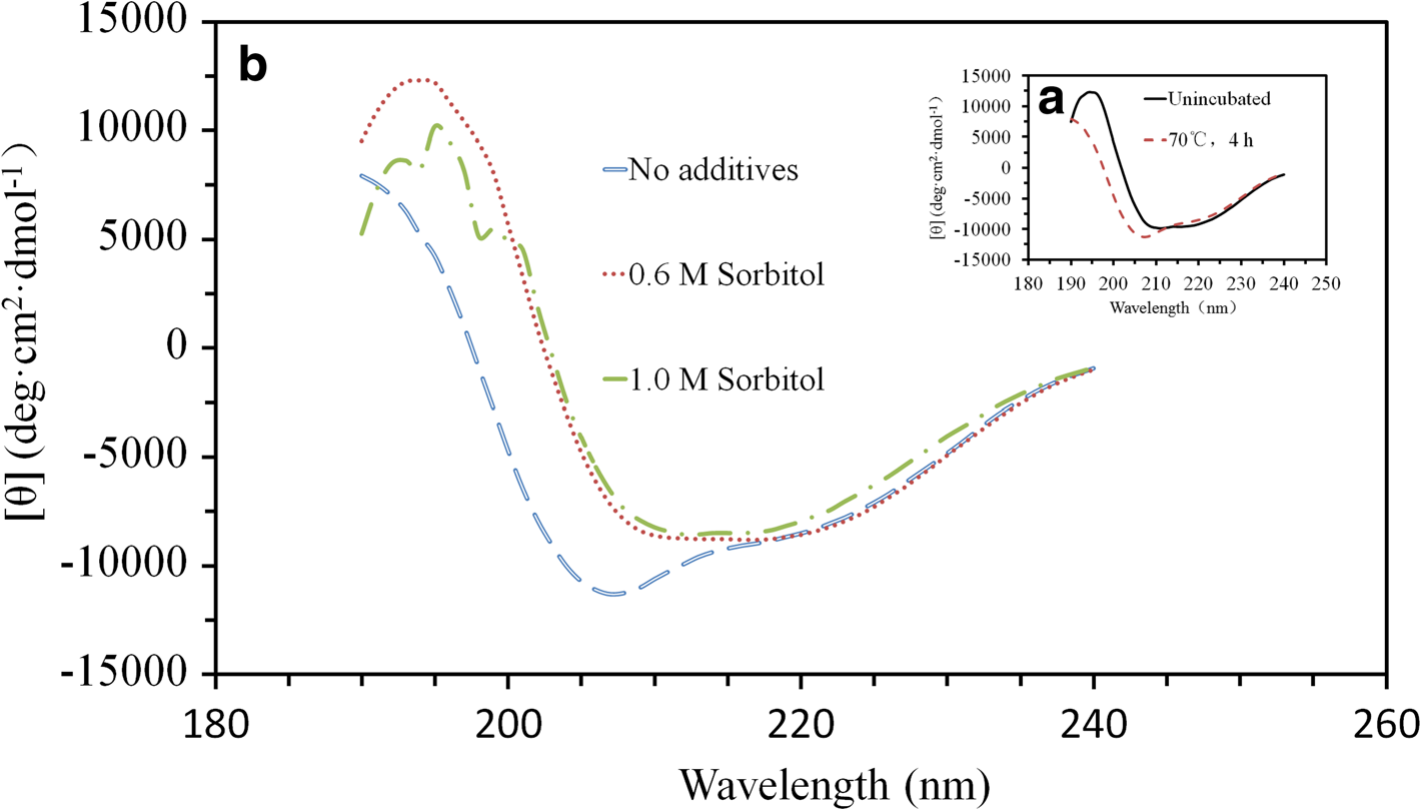
Far-UV CD spectra of α-L-rhamnosidase. **a** Without sorbitol before and after incubation at 70 °C for 4 h. **b** Incubated at 70 °C for 4 h with different concentrations of sorbitol in 10 mM phosphate buffer at pH 6.5. All spectra were corrected for the signal generated by a protein-free sample

It is known that the CD spectra in far-UV region reflects the secondary structure of protein [49]. To investigate the effect of sorbitol on the structure of α-L-rhamnosidase, CD measurements in the far UV (190–240 nm) were performed to reveal the changes that occurred in α-L-rhamnosidase secondary structure, both in the absence and presence of sorbitol, during incubation at 70 °C for 4 h (Fig. 4). Dichroweb was used to convert these CD spectra into the relative contributions of the secondary structural elements (α-helix, β-sheet, turn, and random coil) to the overall structure of α-L-rhamnosidase (Table 4). The α-helix content of the enzyme decreased substantially after incubation at 70 °C for 4 h, and correspondingly, the random coil content increased substantially (Fig. 4a; Table 4).

**Table 4:** Contents of estimated secondary structures of α-L-rhamnosidase with and without sorbitol before and after incubation

Samples	α-Helix (%)	β-Sheet (%)	Turn (%)	Random coil (%)
Control^a^	30	28.88	20.1	21.12
No additives^b^	22	22.48	23.26	32.27
0.6 M Sorbitol^b^	27.17	29.6	21.21	22.12
1.0 M Sorbitol^b^	27.11	31.28	19.56	22.05

^a^Control, α-L-Rhamnosidase before heating

^b^Samples after heating at 70 °C for 4 h

### Effect of sorbitol on the enzymatic conversion of rutin to isoquercitrin

**Fig. 5:**
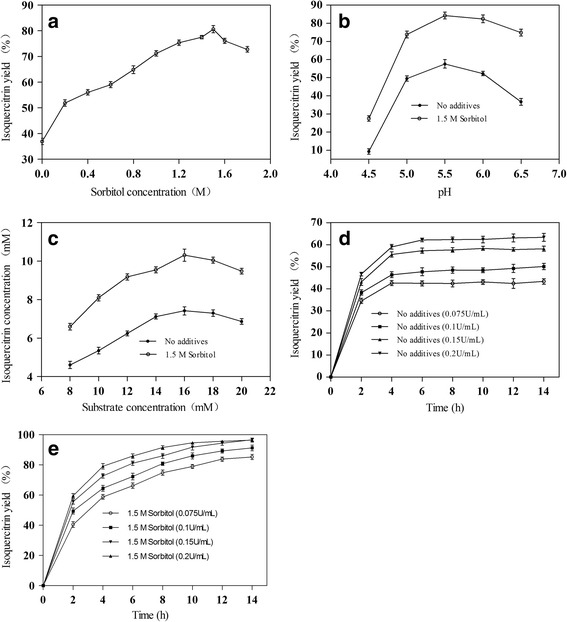
Effect of sorbitol on the enzymatic conversion of rutin to isoquercitrin. **a** Incubated at 70 °C for 4 h with different concentration of sorbitol in disodium hydrogen phosphate-citrate buffer at pH 6.5, 8 mM of rutin, and 0.1 U/mL of α-L-rhamnosidase; (**b**) Incubated at 70 °C for 4 h with 1.5 M of sorbitol in disodium hydrogen phosphate-citrate buffer at pH range from 4.5 to 6.5, 8 mM of rutin, and 0.1 U/mL of α-L-rhamnosidase; (**c**) Incubated at 70 °C for 4 h with different concentration of rutin in disodium hydrogen phosphate-citrate buffer at pH 5.5, 1.5 M of sorbitol, and 0.1 U/mL of α-L-rhamnosidase; (**d**) Incubated at 70 °C for up to 12 h with different enzyme concentration without 1.5 M sorbitol in disodium hydrogen phosphate-citrate buffer at pH 5.5, 16 mM of rutin. **e** Incubated at 70 °C for up to 12 h with different enzyme concentration with 1.5 M sorbitol in disodium hydrogen phosphate-citrate buffer at pH 5.5, 16 mM of rutin. Each value represents the mean of three independent measurements

The enzymatic conversion of rutin to isoquercitrin with sorbitol concentrations of 0.6 M to 1.8 M was tested to investigate the effect of different concentrations of sorbitol. As shown in Fig. 5a, the addition of 0.6 M to 1.8 M of sorbitol did enhance the enzymatic conversion of rutin to isoquercitrin. However, isoquercitrin yield did not increase with an increase in sorbitol concentration. The maximum increase in isoquercitrin yield (41.21%) was observed at 1.5 M of sorbitol because a high sorbitol concentration may decrease the mass transfer coefficient so as to reduce the isoquercitrin yield. Hence, the optimal sorbitol concentration to add to an enzymatic reaction system was calculated to be 1.5 M.

The effect of a buffer pH range (4.5–6.5) on the enzymatic conversion of rutin to isoquercitrin was investigated. As shown in Fig. 5b, the optimal pH of the enzymatic conversion of rutin to isoquercitrin by α-L-rhamnosidase in the absence or presence of sorbitol was 5.5. The addition of sorbitol enhanced the enzymatic conversion of rutin to isoquercitrin in the buffer pH range of 4.5–6.5. Furthermore, the enhancements of sorbitol on enzymatic conversion of rutin to isoquercitrin did not increase with the buffer pH. The addition of sorbitol at a buffer pH of 5.5 increased isoquercitrin yields by 11.5%. Based on this result, the optimal buffer pH for this enzymatic reaction system was calculated to be 5.5.

To investigate the effect of substrate concentrations on the enzymatic conversion of rutin to isoquercitrin, a substrate concentration range from 8 mM to 20 mM was tested. As shown in Fig. 5c, as the concentration of rutin increased, the isoquercitrin concentration increased continuously. However, when the concentration of rutin was higher than 16 mM, the isoquercitrin concentration began to decrease. This result indicates that the substrate concentration plays an important part in the biosynthesis of isoquercitrin. As the substrate concentration increased, substrate inhibition of the reaction gradually became obvious. Therefore, the optimal concentration of rutin for isoquercitrin production by α-L-rhamnosidase was calculated to be 16 mM.

To investigate the effects of enzyme concentration and reaction time on the enzymatic conversion of rutin to isoquercitrin in the absence and presence of sorbitol, a range of reaction times, from 2 h to 10 h, was tested. As shown in Fig. 5, the isoquercitrin yield in the absence and presence of sorbitol almost always reached the maximum after 10 h of reaction. Therefore, the optimal reaction time for the enzymatic conversion of rutin to isoquercitrin was calculated to be 10 h. Compared to the absence of additive, the addition of 1.5 M of sorbitol increased the isoquercitrin yield from 60.01 to 96.43%. Moreover, the enhancements of isoquercitrin yield increased with the increase of reaction time.

## Discussion

The aim of this study was to investigate the effect of sorbitol on the thermostability of α-L-rhamnosidase from *Aspergillus terreus* and enzymatic conversion rutin to isoquercitrin, and to analyze the mechanisms through which the stabilization was achieved. In this study, the increases in thermostability increased as the increase of the sorbitol concentration in the range of 0.2 to 2.0 M at 70 °C. However, the enhancement in thermostability was almost constant in the range of 2.0 to 2.5 M. The greatest increase in stability, a 17.2-fold increase in half-life, was seen at 2.0 M of sorbitol. The results of stability properties indicated addition of 2.0 M sorbitol at temperatures ranging from 60 to 85 °C enhance the thermostability of α-L-rhamnosidase. Furthermore, the results of the kinetics of thermal inactivation can be inferred that addition of 2.0 M of sorbitol serves to maintain the active conformation of enzyme after thermal treatment of high temperature ranging from 65 to 80 °C [50]. The effect of sorbitol on the thermostability of enzymes is not generally predictable. The addition of sorbitol has been reported to be able to increase the thermostability of several enzymes, for example, xylanase from *Thermomonospora sp.* [33], glucose dehydrogenase from *Haloferax mediterranei*. [37] and fungal α-amylase [51]. The results place α-L-rhamnosidase from *Aspergillus terreus* among those enzymes that are stabilized by the addition of sorbitol, and suggest that the addition of sorbitol may make α-L-rhamnosidase from *Aspergillus terreus* suitable for use in industrial processes.

In this study, the addition of sorbitol enhanced the surface hydrophobicity of α-L-rhamnosidase at 70 °C. The result is consistent with the reports of other researchers [46, 52–54], which shows that the sorbitol decreases the direct interaction of water and protein due to the preferential hydration, thereby increasing the thermostability of α-L-rhamnosidase.

In addition, the fluorescence and CD data suggest that the effects of sorbitol on α-L-rhamnosidase are definite. The measurement of intrinsic protein fluorescence is widely used to investigate the changes of tertiary conformation of proteins, because the fluorescence of internal tryptophan residues is particularly sensitive to various perturbations. The α-L-rhamnosidase from *Aspergillus terreus* contains 31 tryptophan residues. The sharp decreased in fluorescence intensity and red-shift of the fluorescence maximum that accompanied heating of the protein to 70 °C, indicating a shift of these residues to a more hydrophilic environment, was reversed by the addition of sorbitol. The extent of the reversal was in proportion to their ability to increase its thermostability. Since these data were relatively easy to obtain and correlated with thermostability, intrinsic fluorescence is considered the simplest predictor of thermostabilizing ability [46]. According to CD data, the addition of different concentrations of sorbitol increased the contents of α-helix and β-sheet, but they decreased the content of random coil (Fig. 4b and Table 4), which indicated the addition of sorbitol to mixture preserved secondary structures in the protein. The result was consistent with the report of other researcher [46], which could explain why addition of sorbitol could improve the thermostability of α-L-rhamnosidase from *Aspergillus terreus*.

Although there have been many reports about improving thermal stabilization of enzymes by polyols, there is no reports about adding a polyol to improve the yield of enzymatic hydrolysis product. In this study, this is the first time to apply sorbitol to improve the thermostability of the enzyme so as to enhance the enzymatic conversion of rutin to isoquercitrin. Addition of sorbitol slightly decreased the value of *K*_*M*_ and *kcat/K*_*M*_, however, compared to the absence of additive, the isoquercitrin yield increased by 1.6 times after addition of 1.5 M of sorbitol, which was attributed to the addition of sorbitol to improve the thermostability of α-L-rhamnosidase. The result indicates that the addition of sorbitol to the reaction mixture makes α-L-rhamnosidase from *Aspergillus terreus* more suitable for use in industrial processes.

## Conclusions

Addition of sorbitol enhanced the thermostability of α-L-rhamnosidase from *Aspergillus terreus* at temperatures ranging from 65 °C to 80 °C. Half-life and activation free energy with addition of 2.0 M sorbitol at 70 °C were increased by 17.2-fold, 8.2 kJ/mol, respectively. Moreover, the isoquercitrin yield increased by 1.6-fold with the addition of 1.5 M of sorbitol at 70 °C. The results suggest that the reaction system by adding sorbitol has great potential to promote enzymatic conversion of rutin to isoquercitrin production.

## Additional file

Additional file 1: Figure S1. The sequence alignment of Rha and MRha. (DOCX 1901 kb)

## Abbreviations

ANS: 8-Anilino1-naphthalenesulfonic acid ammonium salt
BMGY: Buffered glycerol-complex medium
CD: Circular dichroism
HPLC: High performance liquid chromatography
*p*NPR: *p*-Nitrophenyl α-L-rhamnopyranoside
YNB: Yeast nitrogen base
YPD: Yeast extract peptone dextrose medium
