# Supplementary figures and images for: Enhancing the thermostability of α-L-rhamnosidase from Aspergillus terreus and the enzymatic conversion of rutin to isoquercitrin by adding sorbitol
Source: BMC Biotechnol. 2017 Feb 27;17:21. doi: 10.1186/s12896-017-0342-9 (PMC5327507; doi:10.1186/s12896-017-0342-9)

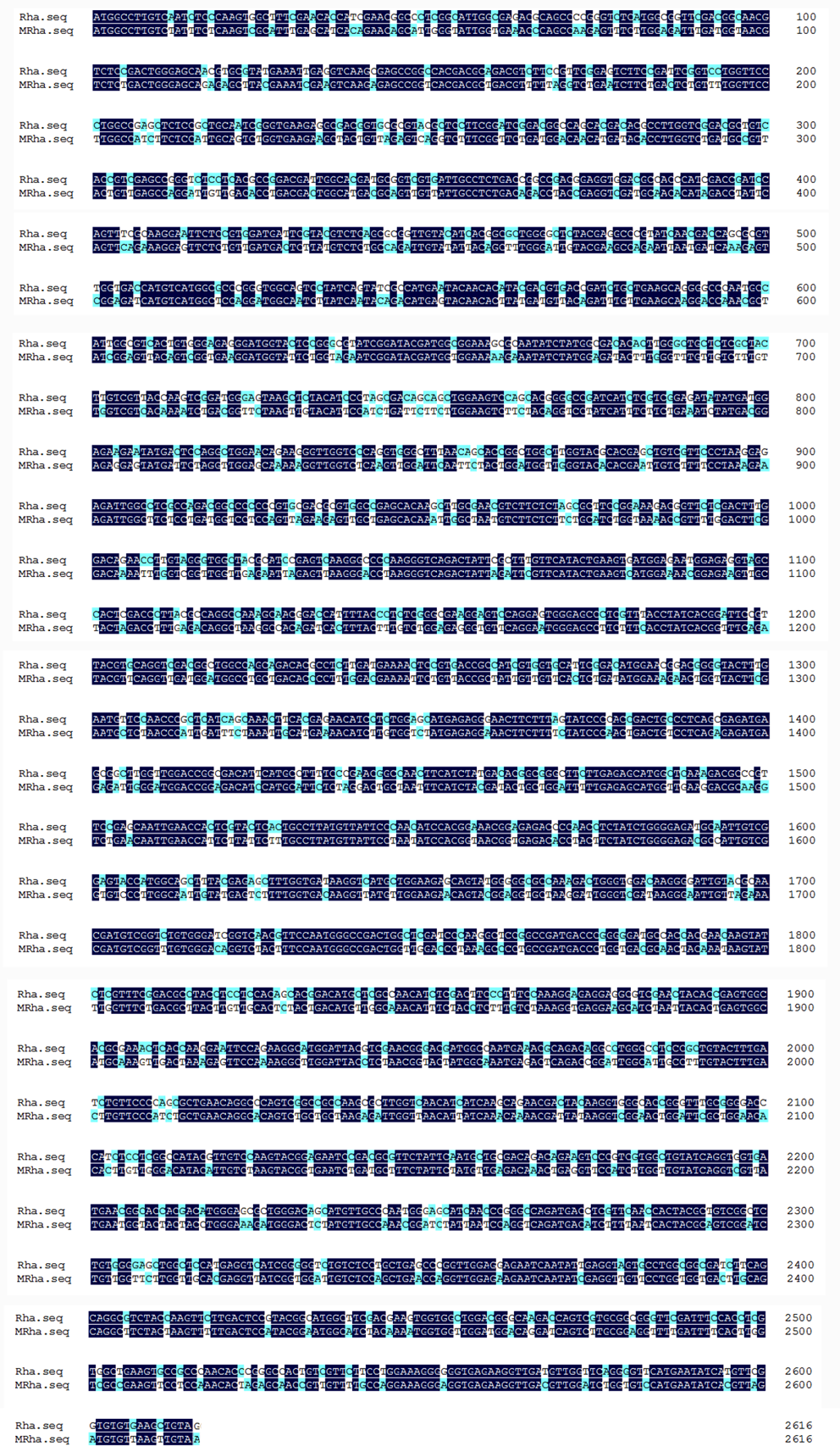


**Figure S1：**The sequence alignment of Rha and MRha.

Supplement: Additional file 1: Figure S1. — The sequence alignment of Rha and MRha. (DOCX 1901 kb) [file 12896_2017_342_MOESM1_ESM.docx]
